# Systematic review on relapse-prevention strategies following successful electroconvulsive therapy for major depressive disorder

**DOI:** 10.1192/bjo.2025.10946

**Published:** 2026-01-14

**Authors:** Jordy J. E. Rovers, Nander T. van Zeijl, Indira Tendolkar, Annemiek Dols, Philip F. P. van Eijndhoven

**Affiliations:** Department of Psychiatry, Radboud University Medical Center, Nijmegen, The Netherlands; Donders Institute for Brain, Cognition and Behaviour, Radboud University, Nijmegen, The Netherlands; Department of Psychiatry, Canisius-Wilhelmina Hospital, Nijmegen, The Netherlands; UMC Utrecht Brain Center, Department of Psychiatry, University Medical Centre Utrecht, Utrecht, The Netherlands

**Keywords:** Electroconvulsive therapy (ECT), major depressive disorder (MDD), relapse prevention, systematic review

## Abstract

**Background:**

Electroconvulsive therapy (ECT) is highly effective for major depressive disorder (MDD), but about 50% of patients relapse within 1 year. A comprehensive review of all potential relapse-prevention strategies is lacking.

**Aims:**

This systematic review aimed to identify, summarise and critically evaluate the available evidence on pharmacological, neuromodulatory, psychological and combination strategies to prevent relapse following successful ECT for MDD.

**Method:**

A systematic review was conducted following the Preferred Reporting Items for Systematic Reviews and Meta-Analyses guidelines (PROSPERO: no. CRD42023392575). We searched PubMed, Embase, PsychInfo and Cochrane Library up to March 2025. Eligible studies included adults (>18 years) with unipolar MDD who responded to acute ECT and were followed for ≥3 months. We included randomised controlled trials (RCTs), cohort studies and case series (over ten cases). Risk of bias and quality were assessed and a narrative synthesis conducted.

**Results:**

A total of 28 studies (*N* = 11 119) were included. Lithium (alone or with antidepressants) was most consistently associated with reduced relapse in 10 studies. Continuation ECT (C-ECT), particularly when combined with pharmacotherapy, also reduced relapse in several RCTs. Evidence for psychotherapy (e.g. cognitive–behavioural therapy) is limited (two studies), warranting further studies. No studies on repetitive transcranial magnetic stimulation or ketamine were found. Study quality varied, with some being underpowered or having used inconsistent definitions of relapse.

**Conclusions:**

Pharmacotherapy with lithium and, separately, C-ECT combined with medication, showed the strongest evidence for relapse prevention following ECT. The evidence base is limited by heterogeneity, small sample sizes and few studies on novel or psychological strategies. More robust clinical studies are needed to identify optimal long-term strategies.

Major depressive disorder (MDD) is a common psychiatric disorder with a lifetime prevalence of 20%.^1,2^ Despite a wide array of available treatments, approximately a third of patients show insufficient response to standard antidepressant treatments.^3^ Electroconvulsive therapy (ECT) is an important option for these patients, and is especially effective in older individuals and in those with severe or psychotic depression.^3–6^ Reported remission rates generally range from 50 to 80%.^4,6^ Although ECT is often viewed as a last-resort treatment, it is more effective in patients with fewer treatment failures and less chronicity, thereby supporting its consideration as a viable option earlier in the treatment algorithm for MDD.^4,6–9^ Despite its efficacy, approximately 50% of patients experience relapse within the first 12 months following successful ECT, most commonly within the first 6 months.^10,11^ This relapsing course imposes a significant burden on patients, their families and society.^12–14^ Notably, similar relapse rates have been reported following successful pharmacotherapy in the STAR*D trial, underscoring the chronic and recurrent nature of MDD.^15^ However, patients receiving ECT typically represent a subgroup with more severe, chronic or otherwise distinct forms of depression. Consequently, relapse prevention in this population may require more intensive or tailored strategies than those recommended for the general MDD population.

Improving long-term outcome following successful ECT is a priority for the field.^16^ Studying predictors of relapse, optimising ECT itself and improving relapse-prevention strategies are means to improve the understanding of relapse and optimise long-term outcome. One study found that patients with psychotic features, greater severity and no prior medication resistance had a lower relapse risk following ECT, although this was limited to those receiving nortriptyline and is not generalisable.^17^ Other, mostly observational, studies examining clinical or treatment factors (e.g. electrode placement, use of medication and comorbidity) have yielded inconsistent results.^18–21^ In the absence of clear evidence identifying which patients are most at risk, relapse prevention remains essential for all individuals who respond to ECT.

In practice, several prevention strategies are available to reduce the risk of relapse in MDD, including pharmacotherapy, neuromodulation, psychotherapy and various combinations of these approaches.^10,22,23^ In a 2013 meta-analysis, Jelovac et al concluded that both continuation pharmacotherapy and continuation ECT (C-ECT), defined as ECT administered regularly during the first 6 months following remission, effectively reduce the risk of relapse, with antidepressant medication shown to reduce relapse rates by approximately 50% compared with placebo.^10^ A subsequent systematic review by Youssef and McCall, which included three RCTs comparing two or more relapse-prevention strategies, found that the combination of C-ECT and continuation pharmacotherapy was the most effective approach.^22^ Similarly, Brown et al supported the efficacy of C-ECT and maintenance ECT (M-ECT), with M-ECT referring to ECT given beyond 6 months to prevent recurrence, particularly when used in conjunction with antidepressants.^24^ More recently, a meta-analysis by Lambrichts et al suggested that the addition of lithium may confer superior relapse prevention compared with regimens without lithium.^25^

Despite these findings, the available evidence remains limited in both scope and quality, because previous reviews often excluded non-randomised designs and focused mainly on biological interventions, with little attention being paid to psychological or novel approaches. A comprehensive reassessment, requiring an integrated evaluation, is therefore warranted. Broadening the inclusion criteria to encompass a wider range of study designs will allow for a more complete synthesis of the existing evidence. Moreover, an integrated evaluation of all major relapse-prevention strategies, including pharmacological, neuromodulatory, psychological and combined approaches, within a single review, will provide a consolidated resource for clinicians and researchers alike. Finally, more recent studies may include emerging interventions, such as repetitive transcranial magnetic stimulation (rTMS) and (es)ketamine which, although commonly viewed as novel avenues in the treatment of difficult-to-treat depression, have not yet been comprehensively assessed in the context of relapse prevention following ECT. Together, these efforts will expand the current evidence base and support more informed clinical decision-making regarding effective relapse prevention following successful ECT for MDD.

## Method

This systematic review adhered to the Preferred Reporting Items for Systematic Reviews and Meta-Analyses guidelines.^26^ A preregistered protocol is available via Preferred Reporting Items for Systematic Reviews and Meta-Analyses (PROSPERO, no. CRD42023392575; registration date 28 January 2023).

### Search strategy and study selection

The electronic databases PubMed, Embase, PsychInfo and Cochrane Library were searched up to 1 March 2025. The search strategy was developed by J.J.E.R. under the supervision of an experienced librarian (Supplement 2 available at https://doi.org/10.1192/bjo.2025.10946). To retrieve as many studies as possible, no limit on publication date was applied. Following the removal of duplicates, titles and abstracts were independently screened by authors J.J.E.R. and N.T.v.Z. using Rayyan (a web-based systematic review software, Rayyan Systems Inc., Cambridge, MA, USA, used on the Windows platform, https://www.rayyan.ai). Articles found to be potentially eligible were selected for full-text screening. Discrepancies in eligibility were discussed between the two reviewers to reach consensus. When consensus could not be reached, a third author (P.F.P.v.E.) was consulted to make the decision. Thereafter, full texts of the selected articles were independently screened by J.J.E.R. and P.F.P.v.E. and discussed until consensus was reached. Initial data extraction from the included studies commenced on 29 April 2025.

### Eligibility criteria

We applied the following eligibility criteria:

- case studies or series (>ten cases), prospective cohort studies, retrospective cohort studies and RCTs;
- participants 18 years of age or above;
- diagnosed with unipolar MDD by clinical judgement or formal diagnostic criteria (e.g. DSM-IV, DSM 5);
- treated with an acute course of ECT for MDD that led to response or remission;
- relapse was defined on the basis of clinical judgement, formally through the use of depression rating scales (e.g. Hamilton Depression Rating Scale (HAM-D)) or using formal diagnostic criteria;
- assessing the efficacy of at least one relapse-prevention strategy following a successful course of ECT for MDD, and with a follow-up of at least 3 months.

The following exclusion criteria were applied:

- primary diagnoses other than unipolar MDD (e.g. bipolar disorder, schizophrenia, schizoaffective disorder, personality disorder, post-traumatic stress disorder);
- full-text articles published in languages other than English or Dutch;
- in studies including mixed diagnostic groups (e.g. both unipolar and bipolar depression), only those in which the unipolar subgroup was analysed separately were eligible for inclusion.

Other diagnostic groups, such as bipolar disorder, were excluded, because this differs from unipolar depression in course, pathophysiology and guideline-recommended relapse-prevention strategies, which could confound interpretation of treatment effects.

### Risk of bias and quality appraisal

Authors J.J.E.R. and P.F.P.v.E. independently assessed the internal validity of the included RCTs using the Risk of Bias 2 (RoB 2) assessment tool for randomised controlled trials.^27^ Appraisal of the methodological quality of all included articles was performed using the Mixed Methods Appraisal Tool (MMAT) version 2018.^28^ J.J.E.R. and P.F.P.v.E. performed appraisal independently and afterwards reached consensus on the conflicting scores.

Due to the narrative nature of the synthesis, formal assessment of publication bias (e.g. funnel plots or Egger’s test) was not feasible. Instead, we searched clinical trial registries (e.g. ClinicalTrials.gov) and grey literature sources (e.g. ResearchGate, Google Scholar, ProQuest) to identify completed but unpublished studies or relevant trial protocols.

### Data extraction

Data were extracted on study design, population characteristics (e.g. age, gender), type and details of the relapse prevention strategy, outcome measures and key results. Because no new RCT data had been identified since the last systematic review by Youssef and McCall, a meta-analysis was not conducted. Instead, the findings were synthesised in a narrative review, structured according to the intervention and level of evidence, in order to provide a comprehensive and integrative overview of the current literature.

### Outcomes

**Fig. 1:**
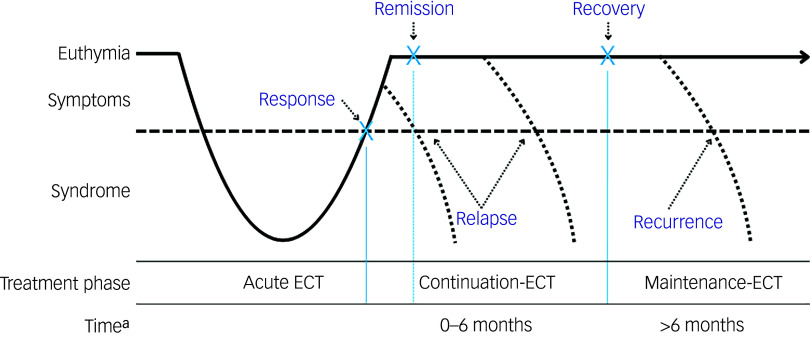
Overview of response, remission, relapse and recurrence in relation to the electroconvulsive therapy (ECT) treatment phase. a. Time represents time since completion of the acute ECT course.

The primary outcome is the relapse rate following a successful (response/remission) course of ECT. Response generally refers to a clinically meaningful reduction in depressive symptoms, whereas remission denotes the virtual absence of symptoms and a return to a euthymic state (Fig. 1).^29^ Different definitions of relapse and recurrence of depression exist. Relapse is defined by Frank et al as indicating a return to full depressive symptomatology beyond the clinical threshold for a diagnosis, but before the individual has reached a full recovery. Recurrence occurs when an individual experiences a new depressive episode after full recovery has been achieved.^30^ In ECT studies, relapse is often heuristically defined as a return to full depressive symptomatology within the first 6 months following response or remission, while recurrence is defined as return of depression after 6 months.^24^ However, in most studies, these terms are often used interchangeably. For the purposes of this review, we wanted to cover both relapse and recurrence within the first 12 months following ECT.

The operationalisation of relapse varied among studies, such as specific cut-off scores on depression severity rating scales, based on clinical judgement and rehospitalisation and/or suicide. All different outcome measure definitions were extracted and included in this review, to ensure the most comprehensive overview of studies into relapse-prevention strategies. The secondary outcomes were time to relapse, risk of relapse (e.g. hazard ratio) and descriptive results.

## Results

### Study selection

**Fig. 2:**
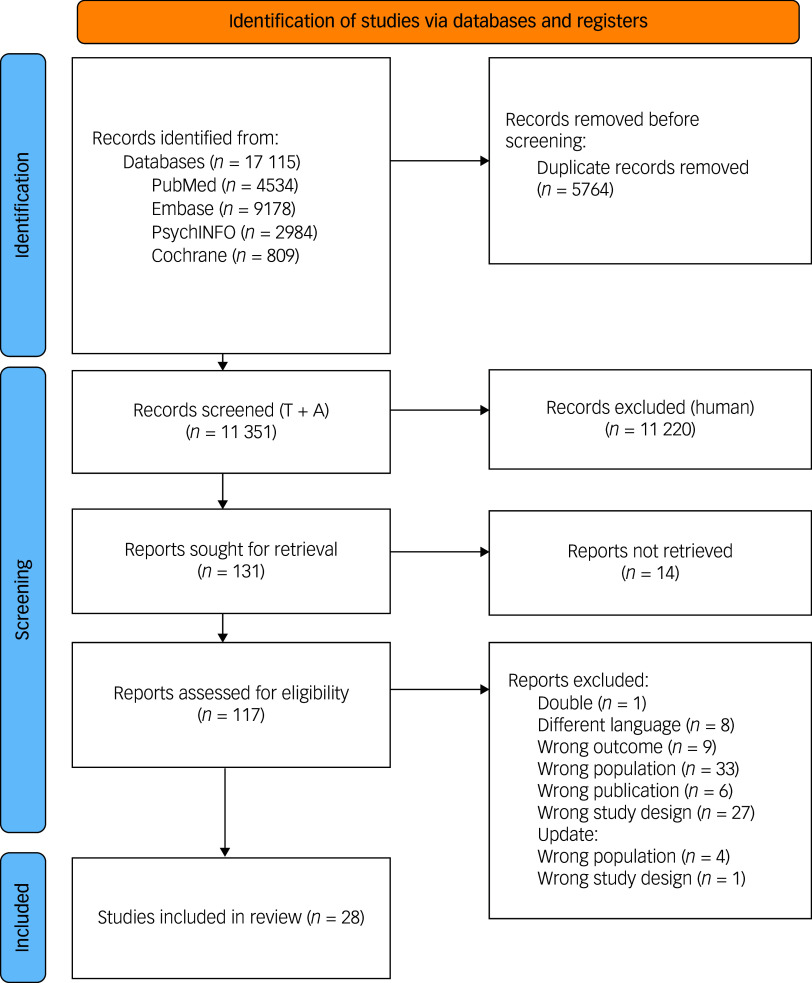
Flow diagram of the study selection process. T + A, title and abstract.

A total of 17 115 records were identified through a literature search up to 1 March 2025. Following removal of duplicates, 11 351 titles and abstracts were screened. Of these, 117 full-text articles were assessed, with 28 meeting the inclusion criteria. The selection process is outlined in Fig. 2.

The literature search identified studies examining pharmacotherapy, neuromodulation, psychotherapy and their combinations as relapse-prevention strategies following successful ECT for MDD. A summary of study characteristics and outcomes is presented in Tables 1 and 2 and Supplement 6; key findings are highlighted below.

**Table 1:** Characteristics of studies on relapse-prevention strategies following successful electroconvulsive therapy (ECT) for major depressive disorder (MDD)

Study	Design	*N*	Population	Mean age (years)	Female gender (%)	Definition of relapse^a^	Electrode placement acute treatment	Measure	Follow-up period (weeks)
Pharmacotherapy									
Coppen et al^31^	RCT	38	MDD recovery	55.1	64	Hospitalisation	NR	HRS	52
Grunhaus et al^32^	RCT	35	MDD responders	60.4	63	Clinical judgement; rating scale threshold	UL, switch to BL	HRSD-17	52
Sackeim et al^33^	RCT	84	MDD remitters	57.4	67	Rating scale threshold	UL, switch to BL	HDRS	24
van den Broek et al^34^	RCT	27	MDD responders	51.4	77	Rating scale threshold	UL; switch to BL	HAM-D 17; CGI-I	24
Yildiz et al^35^	RCT	32	MDD responders	44.2	63	Rating scale threshold	BL	MADRS	18
Martiny et al^36^	RCT	47	MDD remitters	55.3	59	Rating scale threshold	NR	HAM-D 17	25
Sackeim et al^37^	Prospective non-randomised cohort	37	MDD responders	NR	NR	Clinical judgement; rating scale threshold	UL; BL	HAM-D	52
Shapira et al^32^	Prospective non-randomised cohort	19	MDD responders	NR	NR	Rating scale threshold; hospitalisation; change medication	BL	HAM-D	26
Tew et al^38^	Prospective non-randomised cohort	126	MDD responders	57.4	72	Rating scale threshold	NR	HRSD	26
Pluijms et al^39^	Prospective non-randomised cohort	31	MDD remitters	61.2	53	Clinical judgement; rating scale threshold	BL	HRSD; CGI	52
Jelovac et al^40^	Prospective non-randomised cohort	47	MDD remitters	NR	NR	Rating scale threshold; hospitalisation; ECT re-start; (attempted) suicide	BL	HDRS-24	52
Pluijms et al^17^	Prospective non-randomised cohort	34	MDD remitters	63	56	Clinical judgement	BL	HDRS-17	96
Perry et al^41^	Retrospective non-randomised cohort	54	MDD	50.35	57	Clinical judgement; hospitalisation; (attempted) suicide	NR	None	26
Nakajima et al^42^	Retrospective non-randomised cohort	28	MDD remitters	55.5	74	Hospitalisation	NR	None	52
Itagaki et al^43^	Retrospective non-randomised cohort	61	MDD responders	64.9	62	Rating scale threshold; hospitalisation	BL	CGI-I	52
Brus et al^44^	Retrospective non-randomised cohort	7350	MDD	NR	61	Suicide; hospitalisation	UL > BL	None	73
Nordenskjöld et al^21^	Retrospective non-randomised cohort	487	MDD	55	57	Suicide; hospitalisation	NR	None	81
Combination									
Navarro et al^45^	RCT	33	MDD remitters	70.5	64	Clinical judgement; rating scale threshold	BL	HDRS-17	104
Nordenskjöld et al^46^	RCT	56	MDD responders	57	50	Rating scale threshold; hospitalisation; (attempted) suicide	UL	MADRS	52
Kellner et al^47^	RCT	120	MDD remitters	70.6	62	Rating scale threshold; hospitalisation; (attempted) suicide	UL	HAM-D	24
Lin et al^48^	RCT	47	MDD remitters	NR	NR	Rating scale threshold; hospitalisation; (attempted) suicide	BL	HAM-D	12
Martinez-Amaros et al^49^	RCT	37	MDD remitters	68	67	Rating scale threshold	BL	HDRS-21	60
Martinez-Amaros et al^50^	Prospective non-randomised cohort	127	MDD remitters	64.6	63	Clinical judgement	BL	HDRS-21	104
Neuromodulation									
Kellner et al^51^	RCT	184	MDD remitters	57.3	68	Rating scale threshold	BL	HDRS-24	26
Gupta et al^52^	Retrospective non-randomised cohort	38	MDD responders	70.6	89	Hospitalisation	BL; UL	None	208
Al-Wandi et al^53^	Retrospective non-randomised cohort	1873	MDD	62	58	(Re)admission; (attempted) suicide	NR	None	104
Psychotherapy									
Brakemeier et al^54^	RCT	60	MDD responders	61.3	75	Rating scale threshold; hospitalisation	UL, switch to BL	HDRS-24	52
Carstens et al^55^	Prospective non-randomised cohort	8	MDD responders	52.5	63	NR	UL	MADRS	24

RCT, randomised controlled trial; NR, not reported; HRS, Hamilton Rating Scale; UL, unilateral; BL, bilateral; HRSD/HAM-D/HDRS, Hamilton Rating Scale for Depression 17, 21 and 24 items versions; CGI-I, Clinical Global Impressions – improvement scale; MADRS, Montgomery Åsberg Depression Rating Scale.

a. Detailed study-specific definitions are provided in Supplementary Table 6.

**Table 2:** Outcomes of studies on relapse-prevention strategies following successful electroconvulsive therapy (ECT) for major depressive disorder (MDD)

Study	Design	Definition of relapse^a^	Intervention	Comparison	Intervention–relapse (%)/time to relapse (weeks/months)	Comparison–relapse (%)/time to relapse (weeks/months)	Effect
Pharmacotherapy							
Coppen et al^31^	RCT	Hospitalisation	Lithium (0.8–1.2)	Placebo	NR	NR	S
Grunhaus et al^32^	RCT	Clinical judgement; rating scale threshold	Fluoxetine + melatonine	Fluoxetine + placebo	25%	33%	NS
Sackeim et al^33^	RCT	Rating scale threshold	Nortriptyline or nortriptyline + lithium	Placebo	60% (nortriptyline), 39% (nortriptyline + lithium)	84%	NR
van den Broek et al^34^	RCT	Rating scale threshold	Imipramine	Placebo	18%	80%	S
Yildiz et al^35^	RCT	Rating scale threshold	Sertraline early or late	Placebo	14.3%/14 weeks (sertraline early); 31.3%/12.3 weeks (sertraline late)	66.7%/6.33 weeks	NR
Martiny et al^36^	RCT	Rating scale threshold	Escitalopram 10 mg, 20 mg, 30 mg	Nortriptyline 100 mg	45% (10 mg); 36% (20 mg); 50% (30 mg)	14%	NS
Sackeim et al^37^	Prospective non-randomised cohort	Clinical judgement; rating scale threshold	Adequate pharmacotherapy	None	NR	N/A	S
Shapira et al^32^	Prospective non-randomised cohort	Rating scale threshold; hospitalisation; change medication	Lithium	None	26%	N/A	NR
Tew et al^38^	Prospective non-randomised cohort	Rating scale threshold	Protocol treatment (nortriptyline or nortriptyiline + lithium)	Usual care	60% (nortriptyline), 39% (nortriptyline + lithium)	51%	NS
Pluijms et al^39^	Prospective non-randomised cohort	Clinical judgement; rating scale threshold	Nortriptyline during and after ECT	Placebo during ECT, nortriptyline after	47.1%/34.2 weeks	35.7%/40.2 weeks	NS
Jelovac et al^40^	Prospective non-randomised cohort	Rating scale threshold; hospitalisation; ECT restart; (attempted) suicide	Lithium	None	NR	NR	S
Pluijms et al^17^	Prospective non-randomised cohort	Clinical judgement	Nortriptyline	None	50%/18 weeks	N/A	N/A
Perry et al^41^	Retrospective non-randomised cohort	Clinical judgement; hospitalisation; (attempted) suicide	TCA	Lithium	21%	20%	NS
Nakajima et al^42^	Retrospective non-randomised cohort	Hospitalisation	Medication switch after ECT	No medication switch after ECT	0%	43%	NR
Itagaki et al^43^	Retrospective non-randomised cohort	Rating scale threshold; hospitalisation	Medication	None	48.90%	N/A	NR
Brus et al^44^	Retrospective non-randomised cohort	Suicide; hospitalisation	Lithium	No lithium	0% (suicide), 57% readmission	0.83% (suicide), 57% readmission	S
Nordenskjöld et al^21^	Retrospective non-randomised cohort	Suicide; hospitalisation	Lithium; antipsychotics, benzodiazepines, C-ECT	None	34%	N/A	N/A
Combination							
Navarro et al^45^	RCT	Clinical judgement; rating scale threshold	Nortriptyline + C-ECT	Nortriptyline	6.25%/23 months	47%/16 months	S
Nordenskjöld et al^46^	RCT	Rating scale threshold; hospitalisation; (attempted) suicide	Pharmacotherapy + C-ECT	Pharmacotherapy	32%	61%	S
Kellner et al^47^	RCT	Rating scale threshold; hospitalisation; (attempted) suicide	ECT + venlafaxine + lithium	Venlafaxine + lithium	13.10%/7.5 weeks	20.3%/6 weeks	S
Lin et al^48^	RCT	Rating scale threshold; hospitalisation; (attempted) suicide	Agomelatine early	Agomelatine late	50%/9.4 weeks	39.1%/9.2 weeks	NS
Martinez-Amaros et al^49^	RCT	Rating scale threshold	Pharmacotherapy + C-ECT	Pharmacotherapy	47.1%/13.6 weeks	61.1%/11.6 weeks	NS
Martinez-Amaros et al^50^	Prospective non-randomised cohort	Clinical judgement	Pharmacotherapy + C/M-ECT	Pharmacotherapy	59.1%/61 weeks	48.2%/75 weeks	NR
Neuromodulation							
Kellner et al^51^	RCT	Rating scale threshold	C-ECT	Nortriptyline + lithium	37.1%/9.1 weeks	31.6%/6.7 weeks	NS
Gupta et al^52^	Retrospective non-randomised cohort	Hospitalisation	M-ECT	No M-ECT	NR	NR	S
Al-Wandi et al^53^	Retrospective non-randomised cohort	(Re)admission; (attempted) suicide	M-ECT	No M-ECT	35.40%	41.80%	NS
Psychotherapy							
Brakemeier et al^54^	RCT	Rating scale threshold; hospitalisation	Group-CBT + medication	C-ECT + medication; medication	30%	44%, 44%	S
Carstens et al^55^	Prospective non-randomised cohort	NR	Group-CBT	None	NR	NR	N/A

RCT, randomised controlled trial; NR, not reported; S, significant; NS, not significant; N/A, not applicable; TCA, tricyclic antidepressant; C-ECT, continuation electroconvulsive therapy; M-ECT, maintenance electroconvulsive therapy; CBT, cognitive–behavioural therapy.

Definition of relapse, study-specific criteria used to operationalise relapse.

### Pharmacotherapy

Seventeen studies examined the use of various pharmacological agents for relapse prevention following successful ECT. These included six RCTs, six prospective non-randomised cohort studies and five retrospective cohort studies. The studies compared different pharmacological strategies, either to placebo or among one another. In three RCTs that included a placebo condition, relapse rates ranged from 67 to 84% over follow-up periods of 18–24 weeks in the placebo group.

#### Lithium

The earliest RCT by Coppen et al showed that 38 responders receiving lithium had lower scores on HAM-D at 1-year follow-up following ECT^31^. However, these authors had used a narrow relapse definition (only those episodes requiring admission), initiated randomisation during ECT rather than following remission and reported outcomes as ‘weeks ill’ rather than relapse proportion, which limits comparability with more recent trials. In a landmark RCT, Sackeim et al (*n* = 84 remitters) demonstrated that a combination of nortriptyline and lithium significantly prolonged time to relapse and reduced relapse rates compared with both placebo and nortriptyline monotherapy over a 24-week follow-up period (39% nortriptyline + lithium, 60% lithium and 84% placebo).^33^ In this trial, remission at study entry was defined using strict criteria, which might have resulted in a selected remitter sample that differs from typical clinical populations. The frequent follow-up assessments may also have increased the detection of early symptom return across all treatment arms.

Shapira et al prospectively followed ECT responders treated with lithium monotherapy for 6 months post-ECT, and reported a relapse rate of 26% among 19 patients with unipolar depression, lower than rates typically reported in the literature.^56^ Jelovac et al found that lithium maintenance therapy significantly reduced the risk of relapse following ECT, and that lithium was associated with a lower hazard of relapse despite lower mean lithium blood levels compared with the bipolar group.^40^ In that study, remitters had been on lithium prior to, during and following ECT (until relapse or up to 12 months), and were allowed to use concurrent antidepressant medication, which complicates the interpretation of the specific effect attributable to lithium.

Brus et al, using Swedish national registry data (*n* = 7350), found that patients who collected lithium subscriptions, often together with antidepressants, following ECT had a 16% lower risk of hospital readmission, and no suicides occurred in the lithium group.^44^ Perry et al (*n* = 54) retrospectively compared lithium with tricyclic antidepressants (TCAs) for relapse prevention and found no significant difference in relapse rates over 6 months (20% in the lithium group versus 21% in the TCA group),^41^ but these retrospective data may suffer from ‘bias by indication’ and the study predated standardised diagnostic criteria, which limits comparability with modern RCTs. Using data from a regional Swedish quality register, Nordenskjöld et al (*n* = 487) reported that lithium was associated with reduced relapse risk, as measured by rehospitalisation and suicide.^21^ Although their large sample is a clear strength, registry data do not provide information on actual lithium intake, dosage or adherence, and subtle relapse events that do not lead to hospital contact are not captured, which requires cautious interpretation of the observed protective association.

Finally, in a retrospective chart review, Itagaki et al (*n* = 20 responders) found that lithium may be effective in preventing relapse.^43^ However, the small sample, retrospective design and non-standardised pharmacotherapy substantially limit the interpretability of lithium’s apparent effect. Collectively, these studies support the potential preventive value of lithium, as both monotherapy and an augmenting agent, in reducing relapse following ECT. However, it remains unclear whether lithium is most effective as a stand-alone treatment or in combination with antidepressants, particularly given the methodological limitations of the individual studies.

#### Antidepressants

Van den Broek et al (*n* = 27 responders) conducted an RCT comparing imipramine with placebo and found that the former significantly reduced relapse rates following ECT (80% relapsed on placebo versus 15% in the imipramine group).^34^ In this study, post-ECT HAM-D scores were low (mean 4.9–5.9), the total sample size was smaller (*n* = 27) than intended (*n* = 74) and assessors of the Global Clinical Impression scale were probably unblinded, because they also conducted the HAM-D ratings. These factors may have contributed to the large difference observed between groups. Pluijms et al (*n* = 31 remitters) evaluated the timing of nortriptyline initiation in a prospective follow-up study following a RCT, and found no significant difference in relapse rates between patients who started nortriptyline during the ECT course and those who initiated it subsequently.^39^ In the same cohort, Pluijms et al reported relapse rates of 32% at 6 months, 44% at 12 months and 50% at 24 months, which is considerably higher than in the imipramine trial by van den Broek et al and might be a better representation of current realistic relapse rates.^17^ In a RCT, Martiny et al (*n* = 47 remitters) compared nortriptyline with varying doses of escitalopram and found only a marginal, non-significant advantage for nortriptyline, with overall low relapse rates.^36^ The enrolment rate was lower than planned, although sample size was eventually similar to that in other continuation studies. The required discontinuation of psychotropic medication following randomisation complicates interpretation of the outcome. The potential preventive effect of initiating sertraline early (during or immediately after ECT) was investigated by Yildiz et al (*n* = 32 responders), who found it to be superior to both delayed initiation and placebo in time to relapse.^35^ However, the sample size was small, resulting in a shortage of statistical power to detect significance between two active treatment arms. Nakajima et al (*n* = 28 remitters) reported that switching to an antidepressant with a different pharmacological mechanism (e.g. TCA to selective serotonin reuptake inhibitor (SSRI)) following successful ECT was associated with lower relapse rates, suggesting that continuing with pre-ECT medication may be suboptimal.^42^ They based their conclusions on a rather small and unbalanced sample, (*n* = 7 versus *n* = 21), and the outcomes rely mainly on readmission and a measure of social functioning for schizophrenia. By contrast, Sackeim et al (*n* = 37) reported no difference in relapse risk between responders with or without prior TCA resistance.^37^ Finally, Tew et al (*n* = 126 responders), in a naturalistic follow-up study, found no difference in relapse rates between protocol-guided treatment, defined as structured pharmacotherapy with nortriptyline alone, nortriptyline plus lithium or placebo and treatment as usual, suggesting that high relapse rates may not be solely attributable to the so-called efficacy–effectiveness gap (the difference between ideal trial conditions and real-world clinical practice).^38^ However, the high loss to follow-up (29%) may have resulted in an underestimation of relapse in the usual-care group.

#### Other agents

Several studies have investigated alternative pharmacological strategies beyond the commonly recommended TCA–lithium combination for relapse prevention following ECT. In a RCT, Grunhaus et al (*n* = 35 responders) found no added benefit of melatonin when combined with fluoxetine, compared with fluoxetine plus placebo.^32^ However, they reported relatively low relapse rates in both groups. Although patients were clinically followed for 1 year following ECT, relapse was formally evaluated only at the 3-month time point. This shorter effective assessment window, together with the small sample size, may have contributed to the low observed relapse rates. Using data from a regional Swedish quality register, Nordenskjöld et al (*n* = 487) reported that antipsychotics and benzodiazepines were not associated with reduced relapse risk, as measured by rehospitalisation and suicide.^21^ These findings should be interpreted in light of the inherent strengths and limitations of registry data, as discussed above. In a retrospective chart review, Itagaki et al (*n* = 61 responders) found that valproate may be effective in preventing relapse, while no such benefit was observed for various antidepressants or second-generation antipsychotics, although the small retrospective sample and heterogeneous treatment limit interpretation.^43^ No additional studies on alternative pharmacological agents, such as (es)ketamine, were identified.

Overall, pharmacological relapse-prevention strategies appear to reduce relapse rates following ECT. Among these, lithium, as either monotherapy or an augmenting agent, has the most robust and consistent evidence for efficacy across various study designs. In contrast, evidence for antidepressants remains limited in both quantity and methodological quality.

### Neuromodulation

#### C-ECT/M-ECT

Four studies, one RCT and three retrospective cohort studies, have examined the role of C-ECT and M-ECT in relapse prevention. In a well-designed RCT, Kellner et al (*n* = 184 remitters) compared C-ECT (10 sessions over 6 months following bilateral-index ECT) with a pharmacological strategy of nortriptyline plus lithium.^51^ Relapse rates did not differ significantly between the two groups (C-ECT, 37.1%; nortriptyline plus lithium, 31.6%), suggesting comparable efficacy. Notably, both groups showed substantially lower relapse rates than placebo or nortriptyline monotherapy, as seen in studies such as Sackeim et al, supporting the effectiveness of C-ECT and nortriptyline plus lithium as a relapse-prevention strategy.^33^ However, data from a regional Swedish quality register reported by Nordenskjöld et al (*n* = 487) found no association between C-ECT (mean 12 ± 15 sessions) and reduced relapse risk, as indexed by psychiatric hospitalisation or suicide, in a more chronically ill subpopulation.^21^ Similarly, a Swedish population-based register study by Al-Wandi et al (*n* = 1873) found that M-ECT (defined as receiving ECT withing 14 days following discharge) in patients with psychotic unipolar depression was not significantly associated with reduced risk of readmission or suicide in the full sample.^53^ However, in patients over 65 years of age, M-ECT was associated with a significantly lower risk, suggesting age-dependent benefits. These registry findings should be interpreted with caution, because registry data capture only severe relapse events and do not record actual symptom return, which may mask more subtle differences between treatment strategies and could partly explain the discrepancy with the effects of C-ECT reported in clinical trials. Finally, M-ECT, defined as ECT to prevent recurrence (following 6 months of remission) with no fixed end-point, was suggested to play a role in reducing both the rate and duration of hospital stay in a small retrospective cohort study by Gupta et al (*n* = 38) in 19 responders, and in a like-wise comparison group receiving other maintenance therapies.^52^ Overall, C-/M-ECT may be effective, especially in older adults.

#### rTMS

No studies were identified that examined rTMS as a relapse-prevention strategy following ECT for unipolar MDD.

### Psychotherapy

Two studies examined psychotherapy as a relapse-prevention strategy following successful ECT for MDD. In a RCT, Brakemeier et al (*n* = 60 responders) compared group cognitive–behavioural therapy (CBT) plus pharmacotherapy with C-ECT (weekly for 4 weeks, biweekly for 8 weeks and monthly for 3 months) plus pharmacotherapy and pharmacotherapy alone.^54^ Sustained response at 6 and 12 months was significantly greater in the group-CBT arm, suggesting that adjunctive group-CBT may be an effective and well-tolerated approach to maintaining ECT response. The continuation ECT arm performed unexpectedly poorly. This might be due to a small and highly treatment-resistant sample, fewer C-ECT sessions than other schedules used in studies, substantial dropout in the ECT arm or the absence of standardised nortriptyline–lithium pharmacotherapy, all of which limit interpretability. Carstens et al conducted a small prospective study of eight MDD responders who received group-CBT in addition to individualised pharmacotherapy, C-ECT and supportive psychotherapy.^55^ They observed further reductions in depressive symptoms following group-CBT, indicating a possible benefit in prolonging treatment response. Their sample is, however, too small from which to draw any conclusions. Although the addition of psychotherapy as a relapse-prevention strategy seems a promising and feasible approach, the evidence base is currently insufficient to draw firm conclusions.

### Combination of strategies

A total of six studies were included, comprising five RCTs and one prospective non-randomised cohort study. These studies compared the efficacy of a combined approach involving C-/M-ECT and pharmacotherapy, as opposed to sole reliance on pharmacotherapy. In a RCT by Navarro et al (*n* = 33) involving older remitters with psychotic unipolar depression, the combination of C-ECT (weekly treatment for the first month, every 2 weeks for the following month and then monthly) and nortriptyline was more effective in preventing both relapse and recurrence than nortriptyline monotherapy.^45^ However, the study sample was limited to older patients with psychotic depression and each group had four drop-outs, substantially reducing the sample size and resulting in imprecise estimates and limited generalisability. Similarly, Nordenskjöld et al (*n* = 56 responders) conducted a RCT comparing a range of pharmacotherapy strategies with and without C-ECT (weekly for 6 weeks and thereafter every 2 weeks for 46 additional weeks: a total of 29 ECTs for the full year), and found significantly lower relapse rates in the combined treatment group (32 versus 61%).^46^ Nevertheless, interpretation is limited by early termination of the trial, absence of blinded assessments, heterogeneous pharmacotherapy and broadened inclusion criteria that allowed the entry of patients with Montgomery–Åsberg Depression Rating Scale (MADRS) scores <15.

Kellner et al (*n* = 120 remitters) reported favourable outcomes when adding C-ECT to venlafaxine and lithium. Relapse rates at 6 months were lower in the combined group (13.3%) compared with pharmacotherapy alone (20.3%).^47^ In that study, C-ECT was delivered using the symptom-titrated, algorithm-based longitudinal ECT (STABLE)) protocol, starting with four fixed sessions following remission followed by individualised sessions. Due to the complex study scheme used, the authors noted that sampling bias was introduced due to the unwillingness of severely depressed patients to participate. Also, the reported low relapse rates in both groups were probably due to the strictly defined remitter sample, combined venlafaxine–lithium treatment in all patients and intensive STABLE monitoring, which was the primary aim of the study. Nevertheless, it supports the benefit of continuing ECT post-remission rather than abrupt discontinuation. In a smaller RCT by Martínez-Amorós et al (*n* = 37 remitters), relapse rates were lower in the group given C-ECT (weekly for 4 weeks (4 sessions), every 2 weeks for 2 months (4 sessions), and monthly for 6 months (6 sessions), up to a total of 14 sessions in 9 months) plus pharmacotherapy (35%) than in the pharmacotherapy-only group (61%), although the difference was not statistically significant due to early termination and insufficient power.^49^ In another RCT by Lin et al (*n* = 47 remitters) combining agomelatine with an acute ECT course did not reduce relapse compared with initiation of agomelatine following ECT.^48^ This finding is somewhat difficult to compare with other continuation studies because the follow up period of 3 months was shorter than the typical 6–12 months used in most trials. Finally, Martínez-Amorós et al (*n* = 127 remitters) conducted a prospective cohort study with a 2-year follow-up, reporting high relapse rates in both groups: 59.1% with C-/M-ECT (weekly and then monthly (median of 40 weeks and 13 sessions) plus pharmacotherapy, and 48.2% with pharmacotherapy alone.^50^ This unexpected difference probably reflects selection bias, because C-/M-ECT was preferentially prescribed to patients with more severe illness and greater treatment resistance. These naturalistic outcomes contrast with the lower relapse rates seen in RCTs, and underscore the challenges of long-term relapse prevention in routine care. Overall, the evidence suggests that the addition of C-ECT to pharmacotherapy may enhance relapse prevention compared with pharmacotherapy alone, although variability in study design, power and population size limits definitive conclusions.

### Integrated synthesis of relapse-prevention strategies

An integrated summary of relapse-prevention strategies, their typical clinical sequencing and qualitative evidence strength is provided in Table 3.

**Table 3:** Stepwise approach of relapse-prevention strategies following successful electroconvulsive therapy (ECT) for unipolar depression, with corresponding qualitative evidence grading

Step	Strategy	Evidence strength	Key evidence summary	Comments/practical considerations
1. First-line strategy for all responders	Antidepressant + lithium	Most consistent	17 studies into pharmacotherapy; 2 randomised controlled trials (RCTs) and 5 cohort studies into lithium as monotherapy or combined approach, suggesting beneficial effect in preventing relapse	Requires lithium monitoring; tolerability considerations; more feasible than continuation ECT (C-ECT, therefore recommended as step 1
2. When step 1 fails; high risk or severe relapse	C-ECT + pharmacotherapy	Most consistent	4 RCTs and 1 prospective cohort showing lower relapse rates then pharmacotherapy alone	Logistically demanding and resource-intensive; possible interactions of medication and ECT; may be delivered as tapering ECT
	C-ECT	Emerging	1 RCT shows relapse rates compared with pharmacotherapy; 1 retrospective cohort showing no beneficial effect	Logistically demanding; resource-intensive; may be delivered as tapering ECT
3. Long-term prevention; repeated relapses	Maintenance-ECT	Limited	2 retrospective cohort and 1 prospective cohort studies suggesting potential benefits	Might be more beneficial in patients ≥65 years; resource-intensive and logistically demanding
Adjunct	Psychotherapy (cognitive–behavioural therapy) + pharmacotherapy	Emerging	1 RCT and 1 small prospective cohort showing possible symptom and functioning benefits	Supportive to other strategies; further research warranted
Experimental	Repetitive transcranial magnetic stimulation/(es)ketamine	Preliminary/experimental	Case series; study protocols	Research setting; evidence remains preliminary

RCT, randomised control trial; C-ECT, continuation ECT.

### Appraisal of methodological quality

**Fig. 3:**
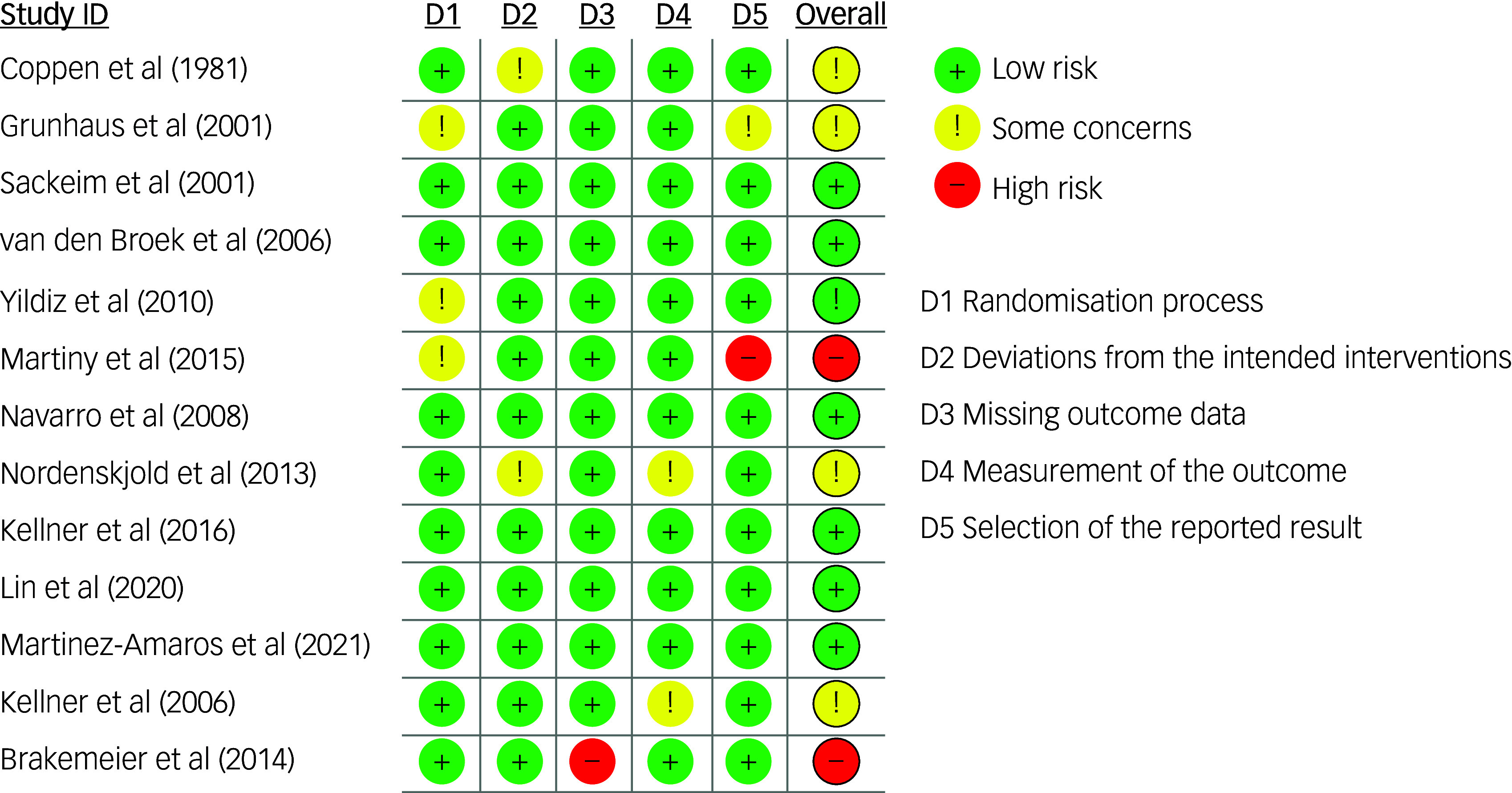
Risk of bias summary (RoB 2) for included randomised controlled trials.

**Fig. 4:**
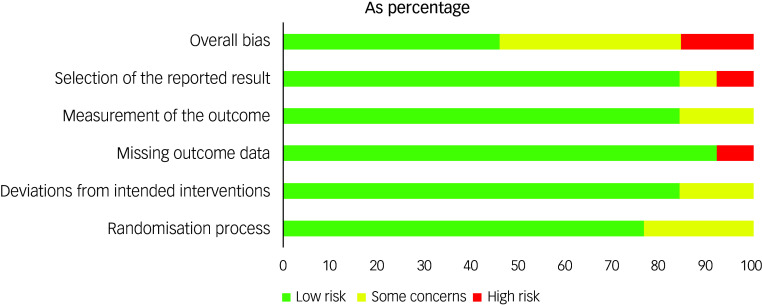
Risk of bias by domain (RoB 2) across included randomised controlled trials.

The MMAT scores varied by study and are reported in Table 4 and Supplement 4. The risk of bias assessment for the RCTs showed that 15% were at high risk for bias, 40% showed some concerns and 45% showed low risk (Figs 3 and 4).

**Table 4:** Quality assessment and risk of bias in studies on relapse-prevention strategies following successful electroconvulsive therapy for major depressive disorder

Study	Design	MMAT score	Risk of bias
Pharmacotherapy			
Coppen et al^31^	RCT	4/5	Some concerns
Grunhaus et al^32^	RCT	4/5	Some concerns
Sackeim et al^33^	RCT	5/5	Low risk of bias
van den Broek et al^34^	RCT	4/5	Low risk of bias
Yildiz et al^35^	RCT	2/5	Some concerns
Martiny et al^36^	RCT	2/5	High risk of bias
Sackeim et al^37^	Prospective non-randomised cohort	5/5	N/A
Shapira et al^32^	Prospective non-randomised cohort	4/5	N/A
Tew et al^38^	Prospective non-randomised cohort	3/5	N/A
Pluijms et al^39^	Prospective non-randomised cohort	3/5	N/A
Jelovac et al^40^	Prospective non-randomised cohort	3/5	N/A
Pluijms et al^17^	Prospective non-randomised cohort	5/5	N/A
Perry et al^41^	Retrospective non-randomised cohort	3/5	N/A
Nakajima et al^42^	Retrospective non-randomised cohort	4/5	N/A
Itagaki et al^43^	Retrospective non-randomised cohort	4/5	N/A
Brus et al^44^	Retrospective non-randomised cohort	4/5	N/A
Nordenskjöld et al^21^	Retrospective non-randomised cohort	4/5	N/A
Combination			
Navarro et al^45^	RCT	4/5	Low risk of bias
Nordenskjöld et al^46^	RCT	2/5	Some concerns
Kellner et al^47^	RCT	5/5	Low risk of bias
Lin et al^48^	RCT	4/5	Low risk of bias
Martinez-Amaros et al^49^	RCT	5/5	Low risk of bias
Martinez-Amaros et al^50^	Prospective non-randomised cohort	5/5	N/A
Neuromodulation			
Kellner et al^51^	RCT	4/5	Some concerns
Gupta et al^52^	Retrospective non-randomised cohort	3/5	N/A
Al-Wandi et al^53^	Retrospective non-randomised cohort	2/5	N/A
Psychotherapy			
Brakemeier et al^54^	RCT	4/5	High risk of bias
Carstens et al^55^	Prospective non-randomised cohort	2/5	N/A

MMAT, Mixed Methods Appraisal Tool; RCT, randomised controlled trial; N/A, not available.

Our search of trial registries identified several relevant studies on relapse prevention following ECT that have not yet been published, including trials investigating ketamine, C-ECT and cognitive remediation (nos NCT02414932, NCT01305707 and NCT04383509). The grey literature revealed no reports of unpublished data contradicting existing literature. Although we found no evidence of large unpublished negative trials, the presence of unreported study outcomes suggests a risk of publication bias, particularly regarding novel interventions.

## Discussion

This systematic review synthesised, summarised and evaluated the scientific literature on relapse-prevention strategies following a successful acute course of ECT for MDD. The findings indicate that pharmacotherapy – particularly lithium, either as monotherapy or in combination with antidepressants – has the strongest evidence base and is the most extensively studied. Combination strategies involving C-/M-ECT alongside pharmacotherapy also show promising effects in reducing relapse risk. Psychotherapy, especially CBT, appears to be a feasible and potentially beneficial adjunct, although current evidence remains limited. Novel interventions such as rTMS and ketamine have not yet been (sufficiently) studied in this context. Despite the heterogeneity and methodological limitations of the available studies, the most consistently supported relapse prevention approaches are lithium-based pharmacotherapy and combined pharmacological and C-/M-ECT strategies. There remains a clear need for more rigorous, high-quality research, particularly into novel biological treatments and the role of psychotherapy in sustaining ECT response over the long term.

Current clinical guidelines for relapse prevention in MDD typically recommend a combination of pharmacotherapy, psychotherapy and lifestyle interventions.^57,58^ The main difference between relapse-prevention strategies in the general MDD population and in those patients treated with ECT lies in the severity and treatment resistance of depressive episodes. Patients who undergo ECT have often failed multiple previous treatments and tend to have more severe, chronic or psychotic forms of depression.^4,10^ As such, relapse-prevention strategies in this population might need to be more intensive and at least include pharmacotherapy or M-/C-ECT. Moreover, as seen in the studies included in this review, not all patients achieved remission: a substantial proportion entered the continuation phase following response (often defined as ≥50% reduction in depressive symptoms) alone. Because responders retain residual symptoms and therefore might remain clinically depressed, relapse in the strict sense cannot occur. Instead, the clinical aim shifts towards sustaining response and supporting further improvement towards remission. Importantly, maintaining response is itself a meaningful and often realistic therapeutic goal in this severely ill, difficult-to-treat population.

While SSRIs and serotonin and norepinephrine reuptake inhibitors are strongly recommended in general MDD guidelines for use over at least 6–12 months following remission, their efficacy in the ECT-treated population is unclear, which might be due to the high rates of medication resistance prior to ECT.^7,10^ This may diminish the utility of antidepressants as monotherapy in relapse prevention, although this hypothesis remains largely untested. By contrast, lithium has been consistently associated with a reduction in relapse risk and is the augmentation agent most studied in this context. The severity and chronicity of illness in ECT recipients further support the rationale for extended pharmacological maintenance and the use of augmentative strategies.

Psychotherapies, such as CBT and mindfulness-based cognitive therapy, are frequently recommended in the general guidelines for relapse prevention.^58–60^ These therapies have been shown to significantly reduce relapse rates, especially when combined with pharmacotherapy.^61^ In contrast, evidence for the use of psychotherapy in the ECT population is lacking. This might be due to the assumption that peri- and post-ECT cognitive impairment might impede the effectiveness of psychotherapies, and that the ECT population has a high level of psychotherapy resistance. Nevertheless, changes in mood-regulating areas of the brain due to ECT might create a window of opportunity for change in thinking, feeling and behaviour.^61^ This window of opportunity could be harnessed to address the broad range of predisposing, precipitating and maintaining factors in MDD, including psychological and psychosocial influences that heighten the risk of relapse.^62,63^ When sufficient adaptations to psychotherapy are made, as demonstrated by Brakemeier et al, who modified CBT by increasing the structure, repetition and use of written materials to accommodate post-ECT cognitive difficulties, it may hold potential as a combined relapse prevention strategy for the ECT population.^54^ Further research is needed to explore how strategies from the general MDD population, such as psychotherapy and lifestyle interventions, can be adapted for use in the ECT population to provide more comprehensive and sustainable relapse prevention.

Interestingly, no randomised studies have yet evaluated rTMS or (es)ketamine as relapse-prevention strategies following ECT. This is a significant gap in the literature, given the growing interest in these novel techniques. A single case series^64^ comprising four patients with unipolar MDD addressed the use of rTMS as a relapse prevention strategy following acute-index ECT. During the follow-up rTMS period, all 4 patients were receiving antidepressant medication and demonstrated sustained responses over a follow-up period of 6–13 months. Although anecdotal, these findings underscore the need for systematic research into novel neuromodulatory or pharmacological maintenance strategies in the post-ECT context.

### Implementation barriers

Although relapse prevention is generally recommended, its implementation in clinical practice remains challenging. Access to C-/M-ECT may be limited by logistical constraints, availability and patient preferences. The potential cognitive effects of C-/M-ECT also merit consideration. While acute ECT is known to cause (most often, transient) cognitive side-effects, recent reviews found no consistent evidence of cognitive decline during C-/M-ECT, although data quality is low and results are mixed, particularly in older adults.^65–67^ Evidence is particularly weak, because most studies used the Mini-Mental State Examination to assess potential cognitive decline, which is now regarded as being insufficiently sensitive.^68^ For these reasons, potential cognitive outcomes should be carefully considered, explicitly discussed with patients when C-/M-ECT is being considered and, ideally, monitored throughout the course of treatment, as also recommended during an acute course of ECT.^69^ Adherence to pharmacological continuation treatment and tolerability, particularly for lithium, are additional barriers. Cognitive concerns or fear of ECT may also influence long-term acceptance of ECT or hinder engagement in psychotherapy. Improving outcomes may therefore also require tackling these implementation barriers.

### Strengths and limitations

The findings of this systematic review should, therefore, be interpreted in light of several other limitations of the included studies. First, the variability in relapse definitions across studies, with some relying on clinical judgement and others using standardised scales, makes it difficult to compare outcomes directly. This inconsistency could result in either over- or underestimation of relapse rates. Furthermore, many of the included studies were underpowered, as seen in trials that were prematurely terminated or involved small sample sizes. This lack of statistical power diminishes the confidence in findings, particularly in studies investigating newer therapies or combinations. Most included studies involved relatively older patient samples (mean age typically >55 years), probably reflecting the demographic profile of contemporary ECT populations, but should be taken into account when interpreting the conclusions. Another notable limitation is the lack of recent high-quality RCTs, particularly regarding novel pharmacotherapies and neuromodulation techniques. Many studies relied on retrospective data or non-randomised designs, which may have introduced biases such as selection bias or confounding variables that are not adequately controlled. Studies also differed in the timing of study entry: in most, participants were enrolled immediately after meeting response or remission criteria; some required a 1-week period of sustained remission, and one study allowed up to 3 weeks between the last ECT session and entry. Several studies did not report this variable. Such variation may affect relapse rates, because more stable patients are less prone to early symptom return. Follow-up duration also varied considerably, with some studies reporting outcomes over less than 6 months, making it difficult to assess long-term relapse risk reliably. C-/M-ECT protocols varied in frequency and tapering speed, ranging from fixed schedules to symptom-titrated approaches such as STABLE, which may have contributed to differences in relapse outcomes.^47^ Despite its limitations, this review provides a comprehensive overview of available relapse-prevention strategies, including a wide range of study designs. It highlights established strategies, such as lithium-based pharmacotherapy and C-/M-ECT, while also identifying underexplored areas such as psychotherapy and novel biological treatments.

In addition to the limitations of the included studies, several aspects of the review itself warrant consideration. Although we conducted a comprehensive search across multiple databases and pre-registered our protocol, the review relied on a narrative synthesis due to the heterogeneity of study designs and outcome definitions, precluding a formal meta-analysis. Another limitation concerns variation in inclusion criteria, with some studies enrolling remitters and others responders (Table 1). These divergent entry thresholds create fundamentally different clinical starting points: in responder samples, full symptom resolution has not occurred, meaning that relapse in the strict diagnostic sense cannot be established. As a result, studies may use the term ‘relapse’ to describe heterogeneous clinical phenomena, which complicates comparability across trials and limits the interpretability of reported relapse rates. Although we included both remitter (*n* = 12) and responder (*n* = 12) samples to reflect real-world ECT practice, this population heterogeneity represents a methodological constraint of both the existing literature and this review. The restriction to English- and Dutch-language publications may have led to the omission of relevant studies. Furthermore, despite dual independent screening and risk-of-bias assessment, subjective judgement cannot be fully excluded. Despite these limitations, strengths of the review include a pre-registered protocol, a comprehensive multi-database search and the inclusion of pharmacological, neuromodulatory and psychological strategies within a single synthesis, offering the most up-to-date overview currently available.

### Future directions

Future research should prioritise rigorous, adequately powered trials assessing the efficacy of combined relapse-prevention strategies, especially those integrating psychotherapy with pharmacological or neuromodulatory approaches. Particular attention should be paid to adapting psychotherapies for patients recovering from ECT and exploring the timing, duration and individual tailoring of such interventions. In clinical practice, standardisation of relapse prevention through a combination of pharmacotherapy and C-/M-ECT, specifically gradual tapering of ECT following remission, could optimise long-term outcomes and minimise the risk of relapse. This approach would allow for more individualised treatment plans and better integration of ECT within the broader framework of depression management.

## Supporting information

Rovers et al. supplementary material 1 Rovers et al. supplementary material

Rovers et al. supplementary material 2 Rovers et al. supplementary material

Rovers et al. supplementary material 3 Rovers et al. supplementary material

Rovers et al. supplementary material 4 Rovers et al. supplementary material

Rovers et al. supplementary material 5 Rovers et al. supplementary material

## Data Availability

Data availability is not applicable to this article as no new data were created or analysed in this study.

## Supplementary material

The supplementary material is available online at https://doi.org/10.1192/bjo.2025.10946
