# Supplementary material for: Systematic review on relapse-prevention strategies following successful electroconvulsive therapy for major depressive disorder
Source: BJPsych Open. 2026 Jan 14;12(1):e37. doi: 10.1192/bjo.2025.10946 (PMC12835720; doi:10.1192/bjo.2025.10946)
Supplement: Rovers et al. supplementary material 1 — Rovers et al. supplementary material [file S2056472425109460sup001.pdf]

**PubMed (P + I)**

("Depressive Disorder"[Mesh:NoExp] OR "Depressive Disorder, Major"[Mesh] OR "Depressive Disorder, Treatment-Resistant"[Mesh] OR "Depression"[Mesh] OR "Affective Disorders, Psychotic"[Mesh] OR "Neurotic Disorders"[Mesh] OR depressive[tiab] OR depression*[tiab] OR unipolar[tiab] OR melanchol*[tiab] OR affective-psychos*[tiab] OR psychotic-affective-disorder*[tiab] OR psychotic-mood-disorder*[tiab] OR neurotic[tiab] OR neurosis[tiab] OR neuroses[tiab] OR major-affective-disorder*[tiab] OR major-mood-disorder*[tiab])
AND
("Electroconvulsive Therapy"[Mesh] OR ECT[tiab] OR electroshock*[tiab] OR electric-convulsive[tiab] OR electro-convulsive[tiab] OR electric-shock*[tiab] OR electroconvulsant*[tiab] OR electroconvulsive[tiab] OR electro-shock*[tiab])
AND
("Antidepressive Agents"[Mesh] OR "Antidepressive Agents" [Pharmacological Action] OR "Antimanic Agents" [Pharmacological Action] OR antidepressive[tiab] OR anti-depressive[tiab] OR antidepressant*[tiab] OR anti-depressant*[tiab] OR pharmacotherapy*[tiab] OR "Psychotherapy"[Mesh] OR Psychotherap*[tiab] OR Psycho-therap*[tiab] OR Cognitive behavior*[tiab] OR Cognitive behaviour*[tiab] OR CBT[tiab] OR Mindfulness[tiab] OR MBCT[tiab] OR Interpersonal therap*[tiab] OR Interpersonal treatment*[tiab] OR (("Electroconvulsive Therapy"[Mesh] OR ECT[tiab] OR electroshock*[tiab] OR electric-convulsive[tiab] OR electric-shock*[tiab] OR electroconvulsant*[tiab] OR electroconvulsive[tiab] OR electro-convulsive[tiab] OR electroshock*[tiab]) AND (continu*[tiab] OR maintenance*[tiab])))

**Embase (P + I)**

(Depression/ or major depression/ or treatment resistant depression/ or affective psychosis/ or Neurosis/ or depressive psychosis/ or late life depression/ or depressive.ti,ab,kf. OR depression*.ti,ab,kf. OR unipolar.ti,ab,kf. OR melanchol*.ti,ab,kf. OR affective-psychos*.ti,ab,kf. OR psychotic-affective-disorder*.ti,ab,kf. OR psychotic-mood-disorder*.ti,ab,kf. OR neurotic.ti,ab,kf. OR neurosis.ti,ab,kf. OR neuroses.ti,ab,kf. OR major-affective-disorder*.ti,ab,kf. OR major-mood-disorder*.ti,ab,kf.)
AND
(electroconvulsive therapy/ OR ECT.ti,ab,kf. OR electroconvulsive.ti,ab,kf. OR electric-convulsive.ti,ab,kf. OR electric-shock*.ti,ab,kf. OR electroconvulsant*.ti,ab,kf. OR electroconvulsive-shock*.ti,ab,kf. OR electroshock*.ti,ab,kf. OR electro-shock*.ti,ab,kf.)
AND
(exp antidepressant agent/ or Exp Mood stabilizer/ or antidepressive.ti,ab,kf. OR anti-depressive.ti,ab,kf. OR antidepressant*.ti,ab,kf. OR anti-depressant*.ti,ab,kf. OR pharmacotherapy*.ti,ab,kf. OR Exp psychotherapy/ or Psychotherap*.ti,ab,kf. OR Psycho-therap*.ti,ab,kf. OR Cognitive behavior*.ti,ab,kf. OR Cognitive behaviour*.ti,ab,kf. OR CBT.ti,ab,kf. OR Mindfulness.ti,ab,kf. OR MBCT.ti,ab,kf. OR Interpersonal therap*.ti,ab,kf. OR Interpersonal treatment*.ti,ab,kf. OR ((electroconvulsive therapy/ OR ECT.ti,ab,kf. OR electroconvulsive.ti,ab,kf. OR electroshock*.ti,ab,kf. OR electric-convulsive.ti,ab,kf. OR electric-shock*.ti,ab,kf. OR electroconvulsant*.ti,ab,kf. OR electroconvulsive-shock*.ti,ab,kf.

OR electroconvulsive.ti,ab,kf. OR electro convulsive.ti,ab,kf. OR electroshock*.ti,ab,kf.) AND (continu*.ti,ab,kf. OR maintenance*.ti,ab,kf.)))

**PsycINFO (P + I)**

(Major Depression/ OR exp "Depression (Emotion)"/ OR treatment resistant depression/ OR Affective Psychosis/ OR Neurosis/ OR depressive.ti,ab,id. OR depression*.ti,ab,id. OR unipolar.ti,ab,id. OR melanchol*.ti,ab,id. OR affective-psychos*.ti,ab,id. OR psychotic-affective-disorder*.ti,ab,id. OR psychotic-mood-disorder*.ti,ab,id. OR neurotic.ti,ab,id. OR neurosis.ti,ab,id. OR neuroses.ti,ab,id. OR major-affective-disorder*.ti,ab,id. OR major-mood-disorder*.ti,ab,id.)
AND
(exp "Electroconvulsive Shock"/ OR ECT.ti,ab,id. OR electroconvulsive.ti,ab,id. OR electroshock*.ti,ab,id. OR electric-convulsive.ti,ab,id. OR electric-shock*.ti,ab,id. OR electroconvulsant*.ti,ab,id. OR electroconvulsive-shock.ti,ab,id. OR electro convulsive.ti,ab,id. OR electroshock*.ti,ab,id. OR electro-shock*.ti,ab,id.)
AND
(exp "Antidepressant Drugs"/ OR exp "Mood stabilizers"/ OR Exp Lithium/ or carbamazepine/ or valproic acid/ OR antidepressive.ti,ab,id. OR anti-depressive.ti,ab,id. OR antidepressant*.ti,ab,id. OR anti-depressant*.ti,ab,id. OR pharmacotherapy*.ti,ab,id. OR exp psychotherapy/ OR Psychotherap*.ti,ab,id. OR Psycho-therap*.ti,ab,id. OR "Cognitive behavior*".ti,ab,id. OR "Cognitive behaviour*".ti,ab,id. OR CBT.ti,ab,id. OR Mindfulness.ti,ab,id. OR MBCT.ti,ab,id. OR "Interpersonal therap*".ti,ab,id. OR "Interpersonal treatment*".ti,ab,id. OR ((exp "Electroconvulsive Shock"/ OR ECT.ti,ab,id. OR electroconvulsive.ti,ab,id. OR electroshock*.ti,ab,id. OR electric-convulsive.ti,ab,id. OR electric-shock*.ti,ab,id. OR electroconvulsant*.ti,ab,id. OR electroconvulsive-shock*.ti,ab,id. OR electro convulsive.ti,ab,id. OR electroshock*.ti,ab,id.) AND (continu*.ti,ab,id. OR maintenance*.ti,ab,id.)))

**Cochrane (P + I)**

([mh ^"Depressive Disorder"] OR [mh "Depressive Disorder, Major"] OR [mh "Depressive Disorder, Treatment-Resistant"] OR [mh Depression] OR [mh "Affective Disorders, Psychotic"] OR [mh "Neurotic Disorders"] OR depressive:ti,ab,kw OR depression*:ti,ab,kw OR unipolar:ti,ab,kw OR melanchol*:ti,ab,kw OR affective-psychos*:ti,ab,kw OR psychotic-affective-disorder*:ti,ab,kw OR psychotic-mood-disorder*:ti,ab,kw OR neurotic:ti,ab,kw OR neurosis:ti,ab,kw OR neuroses:ti,ab,kw OR major-affective-disorder*:ti,ab,kw OR major-mood-disorder*:ti,ab,kw)
AND
([mh "Electroconvulsive Therapy"] OR ECT:ti,ab,kw OR electroconvulsive:ti,ab,kw OR electroshock*:ti,ab,kw OR electric-convulsive:ti,ab,kw OR electric-shock*:ti,ab,kw OR electroconvulsant*:ti,ab,kw OR electroconvulsive-shock*:ti,ab,kw OR electro convulsive:ti,ab,kw OR electroshock*:ti,ab,kw OR electro-shock*:ti,ab,kw)
AND

([mh "Antidepressive Agents"] OR [mh "Antimanic Agents"] OR antidepressive:ti,ab,kw OR anti-depressive:ti,ab,kw OR antidepressant*:ti,ab,kw OR anti-depressant*:ti,ab,kw OR pharmacotherapy*:ti,ab,kw OR [mh Psychotherapy] OR Psychotherap*:ti,ab,kw OR Psycho-therap*:ti,ab,kw OR ("Cognitive" NEXT behavior*):ti,ab,kw OR ("Cognitive" NEXT behaviour*):ti,ab,kw OR CBT:ti,ab,kw OR Mindfulness:ti,ab,kw OR MBCT:ti,ab,kw OR ("Interpersonal" NEXT therap*):ti,ab,kw OR ("Interpersonal" NEXT treatment*):ti,ab,kw OR (([mh "Electroconvulsive Therapy"] OR ECT:ti,ab,kw OR electroconvulsive:ti,ab,kw OR electroshock*:ti,ab,kw OR electric-convulsive:ti,ab,kw OR electric-shock*:ti,ab,kw OR electroconvulsant*:ti,ab,kw OR electroconvulsive-shock*:ti,ab,kw OR electro-convulsive:ti,ab,kw OR electroshock*:ti,ab,kw) AND (continu*:ti,ab,kw OR maintenance*:ti,ab,kw)))
