# Supplementary material for: Systematic review on relapse-prevention strategies following successful electroconvulsive therapy for major depressive disorder
Source: BJPsych Open. 2026 Jan 14;12(1):e37. doi: 10.1192/bjo.2025.10946 (PMC12835720; doi:10.1192/bjo.2025.10946)
Supplement: Rovers et al. supplementary material 2 — Rovers et al. supplementary material [file S2056472425109460sup002.docx]

**List of all included papers**

Coppen A, Abou-Saleh MT, Milln P, Bailey J, Metcalfe M, Burns BH, et al. Lithium continuation therapy following electroconvulsive therapy. Br J Psychiatry. 1981;139:284-7.

Grunhaus L, Hirschman S, Dolberg OT, Schreiber S, Dannon PN. Coadministration of melatonin and fluoxetine does not improve the 3-month outcome following ECT. J ect. 2001;17(2):124-8.

Sackeim HA, Haskett RF, Mulsant BH, Thase ME, Mann JJ, Pettinati HM, et al. Continuation pharmacotherapy in the prevention of relapse following electroconvulsive therapy: a randomized controlled trial. Jama. 2001;285(10):1299-307.

van den Broek WW, Birkenhäger TK, Mulder PG, Bruijn JA, Moleman P. Imipramine is effective in preventing relapse in electroconvulsive therapy-responsive depressed inpatients with prior pharmacotherapy treatment failure: a randomized, placebo-controlled trial. J Clin Psychiatry. 2006;67(2):263-8.

Yildiz A, Mantar A, Simsek S, Onur E, Gökmen N, Fidaner H. Combination of pharmacotherapy with electroconvulsive therapy in prevention of depressive relapse: a pilot controlled trial. J ect. 2010;26(2):104-10.

Martiny K, Larsen ER, Licht RW, Nielsen CT, Damkier P, Refsgaard E, et al. Relapse Prevention in Major Depressive Disorder After Successful Acute Electroconvulsive Treatment: a 6-month Double-blind Comparison of Three Fixed Dosages of Escitalopram and a Fixed Dose of Nortriptyline - Lessons from a Failed Randomised Trial of the Danish University Antidepressant Group (DUAG-7). Pharmacopsychiatry. 2015;48(7):274-8.

Sackeim HA, Prudic J, Devanand DP, Decina P, Kerr B, Malitz S. The impact of medication resistance and continuation pharmacotherapy on relapse following response to electroconvulsive therapy in major depression. J Clin Psychopharmacol. 1990;10(2):96-104.

Shapira B, Gorfine M, Lerer B. A prospective study of lithium continuation therapy in depressed patients who have responded to electroconvulsive therapy. Convuls Ther. 1995 Jun;11(2):80-5. PMID: 7552058.

Tew JD, Jr., Mulsant BH, Haskett RF, Joan P, Begley AE, Sackeim HA. Relapse during continuation pharmacotherapy after acute response to ECT: a comparison of usual care versus protocolized treatment. Ann Clin Psychiatry. 2007;19(1):1-4.

Pluijms EM, Kamperman AM, Hoogendijk WJG, van den Broek WW, Birkenhäger TK. Influence of adjuvant nortriptyline on the efficacy of electroconvulsive therapy: A randomized controlled trial and 1-year follow-up. Acta Psychiatr Scand. 2022;145(5):517-28.

Jelovac A, Kolshus E, McLoughlin DM. Relapse following successful electroconvulsive therapy for major depression: a meta-analysis. Neuropsychopharmacology. 2013;38(12):2467-74.

Pluijms EM, Vinther PT, Kamperman AM, Birkenhäger TK. Clinical characteristics associated with relapse 2 years after electroconvulsive therapy for major depression. Acta Psychiatr Scand. 2023;147(6):561-9.

Perry P, Tsuang MT. Treatment of unipolar depression following electroconvulsive therapy. Relapse rate comparisons between lithium and tricyclics therapies following ECT. J Affect Disord. 1979 Jun;1(2):123-9. doi: 10.1016/0165-0327(79)90031-4. PMID: 162494.

Nakajima S, Ishida T, Akaishi R, Takahata K, Kitahata R, Uchida H, et al. Impacts of switching antidepressants after successful electroconvulsive therapy on the maintenance of clinical remission in patients with treatment-resistant depression: a chart review. J ect. 2009;25(3):178-81.

Itagaki K, Takebayashi M, Shibasaki C, Kajitani N, Abe H, Okada-Tsuchioka M, et al. Factors associated with relapse after a response to electroconvulsive therapy in unipolar versus bipolar depression. J Affect Disord. 2017;208:113-9.

Brus O, Cao Y, Hammar Å, Landén M, Lundberg J, Nordanskog P, et al. Lithium for suicide and readmission prevention after electroconvulsive therapy for unipolar depression: population-based register study. BJPsych Open. 2019;5(3):e46.

Nordenskjöld A, von Knorring L, Engström I. Predictors of time to relapse/recurrence after electroconvulsive therapy in patients with major depressive disorder: a population-based cohort study. Depress Res Treat. 2011;2011:470985.

Navarro V, Gastó C, Torres X, Masana G, Penadés R, Guarch J, et al. Continuation/maintenance treatment with nortriptyline versus combined nortriptyline and ECT in late-life psychotic depression: a two-year randomized study. Am J Geriatr Psychiatry. 2008;16(6):498-505.

Nordenskjöld A, von Knorring L, Ljung T, Carlborg A, Brus O, Engström I. Continuation Electroconvulsive Therapy With Pharmacotherapy Versus Pharmacotherapy Alone for Prevention of Relapse of Depression: A Randomized Controlled Trial. The Journal of ECT. 2013;29(2).

Kellner CH, Husain MM, Knapp RG, McCall WV, Petrides G, Rudorfer MV, et al. A Novel Strategy for Continuation ECT in Geriatric Depression: Phase 2 of the PRIDE Study. Am J Psychiatry. 2016;173(11):1110-8.

Lin CH, Yang WC, Chen CC, Cai WR. Comparison of the efficacy of electroconvulsive therapy (ECT) plus agomelatine to ECT plus placebo in treatment-resistant depression. Acta Psychiatr Scand. 2020;142(2):121-31.

Martínez-Amorós E, Cardoner N, Gálvez V, de Arriba-Arnau A, Soria V, Palao DJ, et al. Can the Addition of Maintenance Electroconvulsive Therapy to Pharmacotherapy Improve Relapse Prevention in Severe Major Depressive Disorder? A Randomized Controlled Trial. Brain Sci. 2021;11(10).

Martínez-Amorós E, Cardoner N, Soria V, Gálvez V, Menchón JM, Urretavizcaya M. Long-term treatment strategies in major depression: a 2-year prospective naturalistic follow-up after successful electroconvulsive therapy. J ect. 2012;28(2):92-7.

Kellner CH, Knapp RG, Petrides G, Rummans TA, Husain MM, Rasmussen K, et al. Continuation electroconvulsive therapy vs pharmacotherapy for relapse prevention in major depression: a multisite study from the Consortium for Research in Electroconvulsive Therapy (CORE). Arch Gen Psychiatry. 2006;63(12):1337-44.

Gupta S, Tobiansky R, Bassett P, Warner J. Efficacy of Maintenance Electroconvulsive Therapy in Recurrent Depression: A Naturalistic Study. The Journal of ECT. 2008;24(3).

Al-Wandi A, Landén M, Nordenskjöld A. Electroconvulsive therapy in the maintenance phase of psychotic unipolar depression. Acta Psychiatr Scand. 2024;150(3):148-59.

Brakemeier EL, Merkl A, Wilbertz G, Quante A, Regen F, Bührsch N, et al. Cognitive-behavioral therapy as continuation treatment to sustain response after electroconvulsive therapy in depression: a randomized controlled trial. Biol Psychiatry. 2014;76(3):194-202.

Carstens L, Hartling C, Aust S, Domke AK, Stippl A, Spies J, et al. EffECTively Treating Depression: A Pilot Study Examining Manualized Group CBT as Follow-Up Treatment After ECT. Front Psychol. 2021;12:723977.
